# Iranian version of modified polycystic ovary syndrome health-related quality of Life questionnaire: Discriminant and convergent validity

**Published:** 2013-09

**Authors:** Fatemeh Bazarganipour, Saeide Ziaei, Ali Montazeri, Fatemeh Foroozanfard, Soghrat Faghihzadeh

**Affiliations:** 1 *Department of Reproductive Health and Midwifery, Faculty of Medical Science, Tarbiat Modares University, Tehran, Iran.*; 2 *Mental Health Research Group, Health Metrics Research Center, Iranian Institute for Health Sciences Research, ACECR, Tehran, Iran.*; 3 *Department of Obstetrics and Gynecology, Kashan University of Medical Sciences, Kashan, Iran.*; 4 *Faculty of Medical Sciences, Zanjan University of Medical Sciences, Zanjan, Iran.*

**Keywords:** Questionnaire, Polycystic ovary syndrome, Quality of life

## Abstract

**Background:** A preliminary report indicated that the Iranian version of modified polycystic ovary syndrome health-related quality of life questionnaire (MPCOSQ) is a valid measure of health-related quality of life (HRQOL) in PCOS patients. Accordingly, the Iranian version of MPCOSQ was subjected to further psychometric analyses among a different sample of patients with PCOS.

**Objective: **To examine discriminant and convergent validity of the Iranian version of MPCOSQ.

**Materials and Methods:** This was a cross sectional study of 200 women with PCOS that was carried out in two private gynecology clinics in Kashan, Iran. Discriminant validity was assessed using the known groups comparison. Convergent validity was evaluated by assessing the correlation between similar content on the MPCOSQ and the SF-36.

**Results: **The mean scores for the MPCOSQ showed that women rated lowest on the infertility and menstrual subscales indicating worst health in these dimensions. The results from the SF-36 questionnaire indicated that emotional and vitality domains were the areas of poorest health. Known groups comparison showed that the MPCOSQ differentiated well between sub-groups of women who differed in PCOS specific symptoms, lending support to its discriminant validity. Convergent validity was assessed and as expected a good positive correlation was found between related subscales of the two instruments.

**Conclusion:** The MPCOSQ has now been extensively tested in Iran and can be considered for using as an outcome measure in future outcome studies in this population.

This study extracted from Ph.D. thesis. (Fatemeh Bazarganipour)

## Introduction

Measuring health-related quality of life is an important issue for patients who suffer from chronic diseases. This is particularly the case in patients who experiencing polycystic ovary syndrome (PCOS). The disease and symptoms linked with PCOS, including amenorrhea, oligomenorrhea, hirsutism, obesity, infertility, anovulation and acne, can lead to a significant deterioration in quality of life (QOL), cause mood disturbances including symptoms of depression, marital and social maladjustment and impair sexual functioning (1). The PCOS health-related quality of life questionnaire (PCOSQ) is among well-developed disease specific instruments that was developed by Cronin *et al* (2). 

It contains 26 items that includes five PCOS-related measures i.e. emotional disturbances, hirsutism, weight difficulties, infertility and menstrual difficulties. It has good reliability, but its validity showed controversial results due to absence of measuring acne (3-5). Thus, the PCOSQ was modified by Barnard *et al *and four questions were added to it in order to evaluate issues associated to acne (6). In our previous study, we reported on initial reliability and validity of the Iranian version of the MPCOSQ (7). Our findings, showed a six-factor solution for the MPCOSQ including emotional disturbance, weight, infertility, acne, menstrual difficulties and hirsutism. However, Barnard *et al* found that the menstrual factor could be divided into menstrual symptoms and menstrual predictability. 

We found a good reliability for the instrument (Cronbach’s alpha ranging from 0.71-0.92), but confidence in the use of an instrument for measuring HRQOL requires strong evidence of its measurement properties. Accordingly, the Iranian version of MPCOSQ was subjected to further psychometric analyses among a different sample of patients with PCOS. To the best of our knowledge, this is the first study that reports on following psychometric properties of the MPCOSQ: testing of discrimination and convergent validity by assessing how the SF-36 and the MPCOSQ measure the same concept. 

It was hoped that the findings from this study might contribute to the exiting literature and help both researchers and health professionals to use the questionnaire in order to improve women’ health.

## Materials and methods

**Design and data collection**

This was a cross sectional study of women with PCOS who attended Infertility center of Shahid Beheshti Hospital and a private gynecology clinic in Kashan, Iran from May to October 2012. The Ethics Committee of the Tarbiat Modares University approved the study. The method of sampling was convenience sampling. All patients gave informed consent. Patients with confirmed diagnosis of PCOS were invited to participate in the study. 

After explaining the study objectives, written consent was obtained from each patient and they were requested to complete the study questionnaires. Patients were eligible if they met each of the following criteria: being 15-40 years old; married; not having non-classic adrenal hyperplasia, thyroid dysfunction and hyperprolactinemia; Iranian; not having problems in speaking or listening; not having previous psychiatric diagnoses or using of psychiatric medications including antidepressants; not taking any prescription medication (except allergy medications and occasional pain medications) for at least three months before entering the study; having two of the following Rotterdam diagnostic criteria:

1) polycystic ovaries visualized on ultrasound scan (presence of 12 follicles or more in one or both ovaries and/or increased ovarian volume i.e., >10 ml), 

2) clinical signs of hyperandrogenism (hirsutism score based on hirsutism score greater than 7 or obvious acne) and/or an elevated plasma testosterone (testosterone >2.0 nmol/l), 

3) having an interval between menstrual periods >35 days and /or amenorrhea, defined as the absence of vaginal bleeding for at least 6 months (i.e.199 days) (8).

**Measures**

**a) The SF-36**

The Short Form Health Survey (SF-36) is “a well-known generic HRQOL measure and includes eight subscales: physical functioning (PF), role limitations due to physical problems (RP), bodily pain, general health perception (GH), social functioning (SF), role limitations due to emotional problems (RE), vitality (VT), and mental health (MH). The score for each subscale range from 0-100 where higher scores indicate better conditions. The Iranian version of SF-36 showed a good validity and reliability” (9). 

**b) MPCOSQ**

The MPCOSQ include 30 questions from six HRQOL areas or domains: emotional disturbances (8 items), hirsutism (5 items), infertility (4 items), weight (5 items), menstrual (4 items) and acne (4 items). Each item is associated with a seven-point Likert scale, in which a score of 7 suggest no problems or difficulties and 1 indicates maximum HRQOL impairment on that item (2, 6). Psychometric properties of MPCOSQ in Iranian population have been verified (7).

**c) Additional measures**

1. Menstrual history: menstruation intervals during preceding 12 months were asked from all patients and were categorized into <21 days, 21-34 days, 35-60 days, >199 days and changeable.

2. Infertility history: having infertility and its duration was recorded according to case reports.

3. BMI: weight and height were calculated by weight/height squared [kg/m^2^] in all patients.

4. WHR: according to the World Health Organization's recommendation the waist circumference measured at the midpoint between the lower margin of the last palpable rib and the top of the iliac crest. Hip circumference was measured around the widest portion of the buttocks, with the tape parallel to the floor.

5. Body hair: clinical assessment of hirsutism was determined using the Ferriman-Gallwey Scoring System (F/G score). Nine body sites (the upper lip, chin, chest, upper back, lower back, upper abdomen, lower abdomen, arm, and thigh) were graded from 0 (no terminal hair) to 4 (severe hirsutism). Scores can range from 0-36. A score of 7 or above was considered positive for hirsutism (10).

6. Acne: acne was determined by Global Acne Grading System (GAGS). “The GAGS considers six locations on the face and chest/ upper back, with a factor for each location based toughly on surface area, distribution, and density of pilosebaceous units. Each of the six locations is graded separately on 0-4 scale where the higher scores indicate severe conditions. The global score is a summation of all local scores. Score of 1-18 was considered as mild acne, 19-30 as moderate, 31-38 as severe, and above 39 as very severe” (11). 

**d) Socio-demographic status**

The study used years of formal education as a measure of socioeconomic status and it was categorized into five levels: no education, first level (1-5 years), second level (6-9 years), third level (10-12 years) and fourth level (more than 12 years). Studies from Iran showed that education could be a good proxy measure for socioeconomic status for Iranians (12).

**Statistical analysis**

1. Discriminate validity: Discriminate validity indicates how well an instrument differentiates between different groups who differ in some characteristics. In this study, discriminant validity was assessed using known groups comparison to test how well MPCOSQ discriminates between subgroups of the study sample who differed in hirsutism, acne, menstrual irregularities, infertility and weight status.

2. Convergent validity: When assessing convergent validity, one assumes that scales related to the same underlying construct show high correlations. To assess convergent validity, we hypothesized a significant correlation would be found between scales with a similar content on the MPCOSQ and SF-36 (the emotional disturbances subscale of the MPCOSQ with the mental health and role-emotional domains of the SF-36). We also hypothesized that these two domains of the SF-36 would correlate more strongly with the emotional disturbances of the MPCOSQ than any of its other five subscales. Correlation value of 0.4 was considered satisfactory (13).

Descriptive data are presented with means and SD unless otherwise indicated. Statistical analysis was performed using Statistical Package for the Social Sciences 15.0 (SPSS Inc., Chicago, IL, USA). P-values less than 0.05 were considered as significant. 

## Results

**Participants**

In all, 200 women with PCOS were included in the study. The mean age (SD) of patients was 26.49±4.42 years. The majority of women had third level education (44%, n=88). Approximately, one third of the women had clinical features of hirsutism and three-quarters were infertile. The majority of participant have waist/hip ratio of >0.8. Socio-demographic and clinical characteristic of the patients are presented in table I.

**Magnitude** **and type of HRQOL impairment**

The distribution of the MPCOSQ and the SF-36 scores are shown in table II. Women scored lowest on the infertility (3.26) and menstrual (4.04) domains indicating worst health in these dimensions. Acne was the least troubling (5.60). The findings from the SF-36 questionnaire indicated that women rated lower on the role emotional (56.95) and vitality (56.08). 

The highest mean score was evident for physical functioning (75.43), indicating the best area of health as measured by the SF-36. The proportion of patients with minimum or maximum results on the questionnaire is shown in table II. The percentage of respondents who scored at lowest level (i.e. floor effect) and at highest level (i.e. ceiling effect) was small.

**Discriminant validity of the MPCOSQ**

Known groups comparison showed that MPCOSQ discriminated well between women who differed in PCOS specific symptoms, supporting the validity the MPCOSQ (Tables III).

**Convergent validity of the MPCOSQ**

As expected, a good positive correlation was found between related subscales of the two instruments. Emotional disturbances of the MPCOSQ correlated with role emotional on the SF-36 (r=0.35, p<0.01). This correlation was greater than with other subscales for example the weight (r=0. 15, p=0.02), infertility (r=0.32, p<0.01), hirsutism (r=0.13, p=0.05), menstrual (r=0.17, p=0.01) and acne domain (r=0.26, p=0.03). Also, emotional disturbances of the MPCOSQ correlated with mental health on the SF-36 (r=0.49, p<0.01) and this was more strongly correlated than with the other five scales of the MPCOSQ, including infertility (r=0.47, p<0.01), hirsutism (r=0.25, p<0.01), weight (r=0.25, p<0.01) and menstrual problems (r=0.27, p<0.01) and acne (r=0.21, p<0.01).

**Table I:** Socio-demographic and clinical characteristic in PCOS patients

Age (years)*					26.49±4.42
Education (years) **					
				1-5	22 (11)
				5-9	36 (18)
				10-12	88 (44)
				>12	54 (27)
Hirsutism (FG>7) **					59 (29.5)
Acne **					
		Mild			120 (60)
		Moderate			21 (10.5)
		Severe			2 (1)
Interval between menstruation (days)**					
			<21		6 (3)
			21-34		73 (36.5)
			35-60		13 (6.5)
			>199		20 (10)
			Variable		88 (44)
Having of infertility **					163 (81.5)
BMI (kg/m2)**					
	<25				84 (42)
	25-30				83 (41.5)
	> 30				33 (16.5)
Waist/hip ratio**					
	<0.8				97 (48.5)
	>0.8				103 (51.5)

FG: Ferriman–Gallwey score.

* Mean ±SD.

** N (%).

**Table II:** MPCOSQ and SF-36 scores, floor and ceiling effects

**Scale **	**Subscales**	**Mean (SD)**	**95% CI**	**Minimum (% floor)**	**Maximum (% ceiling)**
MPCOSQ					
	Emotional	4.21 (1.57)	3.99-4.43	0.5	2.5
	Hirsutism	5.24 (1.93)	4.97-5.51	2	3.5
	Acne	5.60 (1.59)	5.37-5.82	1.5	3
	Infertility	3.26 (1.66)	3.03-3.49	0.7	2
	Menstrual	4.04 (1.42)	3.84-4.24	0.5	2
	Weight	4.87 (1.88)	4.60-5.13	2	1
SF36					
	Physical functioning	75.43 (22.46)	72.27-78.58	0	14.5
	Role-physical	61.61 (36.03)	56.56-66.66	14	34
	Bodily pain	59.54 (20.28)	56.70-62.38	0	0
	General health	61.13 (19.59)	58.39-63.88	0	1.5
	Vitality	56.08 (20.48)	53.21-58.95	0	2.5
	Social functioning	60.17 (21.43)	57.17-63.17	0.5	0
	Role-emotional	56.95 (40.41)	51.30-62.60	24.5	38
	Mental health	61.63 (22.10)	58.53-64.73	0	4
	PCS	64.42 (18.44)	61.79-65.05	0	0
	MCS	58.69 (21.43)	55.68-61.70	0	0

PCS: Physical Component Summary.

MCS: Mental Component Summary.

**Table III:** Scores in subscales of MPCOSQ in different groups of PCOS patients

**Groups **		**Domain of MPCOSQ**					
		**Emotional**	**Hirsutism**	**Weight**	**Acne**	**Infertility**	**Menstrual**
Hirsutism score							
	Normal F/G	4.51 (1.56)	5.76 (1.48)	5.17 (1.80)	5.76 (1.62)	3.50 (1.76)	4.29 (1.50)
	F/G>7	3.61 (1.48)	3.51 (1.86)	4.14 (1.92)	5.12(1.62)	2.77(1.41)	3.51 (1.25)
	p-value*	0.49	0.005	0.35	0.74	0.070	0.06
Acne							
	Mild	5.57 (1.21)	5.87 (1.49)	6.200 (1.31)	5.47 (1.60)	4.50 (0.35)	3.97 (1.40)
	Moderate	4.42 (1.64)	5.25 (1.97)	4.91 (2.04)	4.62 (0.88)	3.27 (1.87)	3.91 (1.57)
	Severe	4.06 (1.51)	4.10(1.83)	4.87(1.82)	3.98(1.68)	3.17 (1.55)	2.80 (0.28)
	p-value**	0.26	0.25	0.60	0.001	0.50	0.51
Interval between menstruation (days) ¥							
	Normal	4.31 (1.68)	5.71(1.67)	5.26 (1.68)	5.59 (1.71)	3.57 (1.81)	4.51 (1.46)
	Abnormal	4.16 (1.51)	5.01 (2.02)	4.60 (1.94)	5.58 (1.53)	3.09 (1.56)	3.76 (1.35)
	p-value*	0.32	0.32	0.09	0.69	0.07	0.25
Infertility							
	No	5.57 (1.49)	5.58 (1.81)	5.31 (1.59)	5.77 (1.62)	3.91 (1.95)	4.43 (1.50)
	Yes	4.13 (1.58)	5.17 (1.95)	4.76 (1.93)	5.56 (1.59)	3.11 (1.56)	3.95 (1.40)
	p-value*	0.51	0.44	0.06	0.92	0.008	0.51
BMI (kg/m^2^)							
	< 25	4.14 (1.54)	5.14(2.05)	4.70 (1.64)	5.41 (1.67)	3.21 (1.60)	4 (1.45)
	25-30	4.41 (1.48)	5.40 (1.75)	5.75 (1.71)	5.88 (1.47)	3.51 (1.74)	4.14 (1.53)
	> 30	3.89 (1.81)	5.09 (2.05)	3.02 (1.35)	5.38 (1.62)	2.76 (1.66)	3.88 (1.08)
	p-value**	0.24	0.61	0.000	0.11	0.08	0.64
WHR							
	<0.8	4.21 (1.54)	5.36 (1.57)	5.19 (1.91)	5.91 (1.62)	3.32 (1.93)	4 (1.34)
	>0.8	4.08 (1.61)	5.37 (1.94)	4.47 (1.87)	5.29 (1.71)	3.09 (1.49)	3.98(1.50)
	p-value*	0.65	0.97	0.02	0.06	0.43	0.94

FG: Ferriman-Gallwey score.

Data presented as Mean (SD).

¥ Abnormal means having amenorrhea, oligomenorrhea, polymenorrhea or changeable; normal: the remaining.

* T test.

** ANOVA test.

## Discussion

This is the first study that reports further findings on psychometric properties of the Iranian version of MPCOSQ among a PCOS population. The present study provides support for the discriminant and convergent validity of the MPCOSQ. The results also confirm that PCOS has a considerable negative impact on the HRQOL of women with the condition. Perhaps not surprisingly, infertility and menstruation appeared to be the most significant aspects of the illness that it is in line with our previous study bearing in mind that as indicated earlier we used a different sample for this study (13). 

The clinical characteristics of the sample may explain why the infertility was reported to have the most negative influence on HRQOL. But, if we only pay attention to the prevalence of symptom, the prevalence of acne was about 70% and last domain affected. In addition, PCOS was found affected on all domains in the non-specific SF-36. The psychological domains were most affected by PCOS. In other words, according to the SF-36, PCOS affects women psychologically more than physically. Known-groups comparison indicated that the MPCOSQ subscales were able to make a very well distinction between subgroups of respondents who differed in clinical status, lending support to its discriminant validity. 

The study findings showed that patient with more hirsutism and acne scores, having menstrual irregularities and infertility and more weight, had poorer HRQOL compared to patient without these clinical symptoms. Guyatt *et al *found that PCOSQ scores are correlated weakly with objective measures of hair growth, menstrual cyclicity and hyperandrogenemia. Furthermore, it was found that the proportion of normal menstrual cycles correlated only with the infertility subscale (r=0.17, p<0.01) at baseline but correlated with both the infertility subscale (r=0.27, p<0.01) and menstruation subscale (r=0.24, p<0.01) at 44-week follow-up (3). Barnard *et al* found BMI to be moderately correlated with the PCOSQ Weight subscale (r=-0.60 and r=-0.47, p<0.05) (6). Also, Ching *et al* found BMI to be significantly negatively correlated with all domains on PCOSQ except for the hirsutism subscale (p<0.01) (14).

There were small floor and ceiling effects, in all subscales of the MPCOSQ. Ceiling and floor effects occur when patients record the maximum or minimum health status score on a ratings scale. A lower incidence of ceiling and floor effects also was seen when the disease-specific MPCOSQ was compared with the SF-36. Ceiling and floor effects are more likely with generic instruments because some domains measured were not relevant to the disease process being studied (15). This lower incidence of ceiling and floor effects may result in an instrument that is more responsive to clinical change. Further study of patients with repeated measurements after treatment would be useful to evaluate the responsiveness of this instrument. 

The MPCOSQ subscales moderately correlated with similar subscales of the SF-36 as hypothesized and overall less with the other subscales on the questionnaires. Similarly, Jones *et al* found strong correlations between the emotional disturbances subscale of the PCOSQ and the SF-36 mental health (r=0.62, p<0.01) and role emotional subscales (r=0.49, p<0.01) (4). Coffey *et al* also found significant correlations between the SF-36 mental component summary score and each PCOSQ domain (emotional disturbances: r=0.61, hirsutism: r=0.32, weight: r=0.51, infertility: r=0.49, menstruation: r=0.25) (16). 

The current study has some limitations. The study of patients with PCOS who were attending two private gynecology clinics may limit generalization of the findings to the entire PCOS population. Furthermore, all of the patients in this study were married. There are some items in MPCOSQ related to sex and infertility and thus due to cultural considerations we could not ask these items from single patients. Therefore, the results of the present study have to be interpreted with some care. Further studies using the Iranian MPCOSQ are now needed with regard to larger samples, including community participants, and other regions in Iran. However, further use of MPCOSQ, ideally in a trial with a treatment, would help to establish further measurement properties for the MPCOSQ. Such studies will help to generalize the Iranian version of MPCOSQ. 

In summary, our psychometric results showed that the MPCOSQ has good convergent and excellent discriminate validity. The MPCOSQ has now been extensively tested in Iran and can be used for measuring outcomes studies in the future. 

## Conclusion

The convergent and discriminate validity of the MPCOSQ is supported. The MPCOSQ is a valid outcomes measure for Iranian patients with PCOS.
